# Memoir of the Late Alex. Nasmyth, Esq.

**Published:** 1850-07

**Authors:** Chapin A. Harris


From the Am. Journal of Dental Science.

### MEMOIR OF THE LATE ALEX. NASMYTH, ESQ.

### SURGEON-DENTIST TO HER MAJESTY QUEEN VICTORIA, AND THE ROYAL FAMILY.

BY CHAPIN A. HARRIS, M. D.

It is not only justly due to the memory of talent and worth, and gratifying to surviving friends, but useful and edifying to society, to record striking examples of eminence in life attained by men who had nothing to invest in its concerns but that best of patrimonies, a good education, honorably, intelligently and industriously applied to the arbitration of their fortune and the extention of their fame.

Such eminence was attained through such means by the lamented subject of this memoir; for, alas! it was the wish of the All Wise, in the height of his usefulness and honor, to visit him with disease of body and mind, and consign him to a premature grave. His cherished memory alone remains.

Mr. Nasmyth was a native of Edinburgh, where his father was a respectable clothier, and who died at the advanced age of eighty-seven years. Though the father's worldly means were but small, and the expenses of a large family necessarily very great to him, yet his resolution was to give his children fortunes in their educations, and in that noble determination he succeeded to a wonderful degree. He was an upright and good man, universally esteemed and respected by his fellow-citizens and affectionately beloved by his children. His son, Alexander, (the object of our present observations,) was born in Edinburgh, in the year 1789. He was a man of about five feet eleven inches in height, of a florid complexion, sandy colored hair, rather slender in form, though muscular, and retained a strong Scottish accent; was calm, gentlemanly, and winning in his manners, easy of access to all; of a most liberal disposition, humane and benevolent, and one who, instead of envying the success of others, was ever more ready to assist by money and by counsel, the deserving and the needy who applied to him, than his numerous applicants were to appeal to his sympathies.

Not much requires to be said about Mr. Nasmyth's early life; although the circumstances attending it, form ingredients in his history not without interest, nor to be overlooked. He was put to school at a tender age, and shared in all the advantages of education which the excellent schools of Edinburgh are capable of affording. After passing, with credit to himself as a boy, through the usual scholastic curriculum at the High School at Edinburgh, he was, though not at that time intended to be a professional man, transferred to the University of Edinburgh, where, for a considerable time, he applied himself to the higher and more philosophical branches of study.

He had an uncle and aunt, who, having no children of their own, formed the strongest affection for him, and treated him very much as if he had been their own son. That uncle was an extensive printer in Edinburgh, and subsequently he and Mr. Nasmyth became connected together in the business of paper manufacturing, and wholesale stationers. For some years the prospects of success in this line of life were flattering; but a great depression suddenly took place in the paper trade and business, and the firm sustaining great and unexpected pecuniary losses, through the failure of many of the most eminent publishers, and other persons, the undertaking had to be brought to a close, and Mr. Nasmyth was left to direct his attention to some other pursuit.

During the period he was engaged in this business, he still availed himself of every opportunity, and devoted as much of his time as possible, to scientific researches, and applied himself particularly to the study of Chemistry and Natural Philosophy. He had, moreover, from an early period, enjoyed the warm and hearty friendship of Dr. Thomas Thomson, the present venerable and eminent Professor of Chemistry in the University of Glasgow, (but at that time resident in Edinburgh,) who greatly benefited him by encouraging the natural inclination of his mind to study, and whose friendship Mr.

Nasmyth not only retained in after life, but to the last highly valued.

His younger brother, Mr. Robert Nasmyth, was at this period a Surgeon Dentist in Edinburgh. He had established himself in that profession in 1811; and from his abilities, and unremitting attention to his duties, had soon secured to himself a very extensive practice, and is now, and has for many years past, been the first Surgeon Dentist, in point of skill and eminence in his profession, either in Edinburgh or in Scotland. Mr. Alexander, having witnessed the successful progress of his brother, and possessing that brother's warmest and most affectionate friendship, with his approbation, determined to qualify himself for the practice of the same profession.

Arriving at this resolution, in 1822 he assiduously commenced the study of Dental Surgery. His brother had long been in the habit of benevolently setting apart a portion of every morning, to the bestowment, gratuitously, of his skillful aid to the poor who were afflicted with diseases of the teeth and the adjacent parts of the mouth, and who resorted to him in such numbers, that, for many years, he had, even then, been in the habit of treating annually, no fewer than from 1500 to 1700 cases of that class of people. This necessarily afforded Mr. Alexander an opportunity of seeing and of assisting in the treatment of such cases, and of quickly gaining an extensive insight into Dental Surgery, such as he could not have met with elsewhere, and was a privilege which but very rarely could be expected to fall to the lot of a student.

After having prosecuted his studies, and acquired all the valuable information that was to be obtained in Edinburgh, he proceeded to Paris to gain any additional knowledge that might be had there; to learn the best system of manufacturing mineral teeth, and to acquaint himself with the difference of practice in the two countries. While in Paris he principally confined himself to improvement under the instruction of M. Audibran, and to observing narrowly his mode of practice.

Terminating his sojourn in Paris he returned to Edinburgh, and after remaining a little longer with his brother, taking an active part in his operating room, he directed his steps to that great mart and focus of talent, and of competition for all men, and all commodities—London; and, in the latter end of 1825, commenced the practice of his profession there.

With every possible dilligence he applied himself, not only to the discharge of his professional duties, as soon as he was called upon to do so, but to the cultivation and improvement of his then already considerable acquirements, like all men who feel they have much to learn. He became a practitioner; but he continued to be a student, availing himself of every opportunity to qualify himself to become a member of the Royal College of Surgeons, London. In 1831, he presented himself for examination in that College, and easily passing through it, obtained his diploma, and became one of its members.

He was early discovered to be a man of no ordinary ability, and thoroughly acquainted with the fundamental principles of his profession, which very soon gained him the admiration and approval of the most eminent practitioners in London, who often consulted him, and sought for much of his assistance on nice and difficult points arising in their practice. This was not only highly honorable to him, but tended quickly to introduce him to the notice of the first circles of society, and was therefore extremely encouraging and serviceable to him in that way, and through his meek, courteous, and gentlemanly demeanor to all, and his unceasing attention to his business, a large share of lucrative practice rapidly flowed in upon him, which he never afterwards lost, but which, on the contrary, continued to increase, both in point of extent and respectability, until the hour when his physical and mental energies were both suddenly prostrated.

Owing to what may be called, in his case, an untoward start in early life, by which a great portion of his valuable time was consumed; not having had, in his very young days, the prospect set before him, or the ambition instilled into his mind, of becoming a literary or professional man, and having been cut down at a comparatively early age, when neither he nor his admiring friends had looked forward to a close, so painful and so abrupt, of his valuable exertions ; and recollecting, also, that he was most extensively engaged, and had for years been so, in the practical part of his laboring profession, it is not to be expected that he should have been, when he died, an extensive author upon subjects in themselves requiring a study and research for which any one life-time must be far too short; but still the tree of science has not been left without a twig, nor the archives of literature without fair and valuable contributions from his pen,—though undoubtedly greatly short of what science and mankind would have had to have been grateful to him and to his memory for, had it been consistent with unerring wisdom that his health and life should have been prolonged yet a little longer.

For several years after he began his career in London, he devoted himself to microscopic investigation of the intimate structure of the teeth, but he found his efforts in those investigations greatly thwarthed by the imperfections he detected in the best instruments he could meet with. In 1837 or 1838, he had the Microscopic Observations of Professor Retzius sent to him, with which he was much gratified, in which he took the deepest interest, and which seemed to impart a new impetus to his own zeal. After many exertions, and suggestions of numberless experiments, (for he had great mechanical skill himself,) he suceeeded in procuring a microscope to be made for him, of unsurpassed power and degree of perfection. He collected, also, specimens of the teeth, at every age of almost every animal known to be furnished with a tooth; and specimens of the sculls of all the lower animals, and of all people, at every age, and of every caste and tribe that he could possibly obtain. Thus supplied with an abundance of material so valuable, and means of learning so great, he prosecuted his microscopic investigations with an enthusiasm and nicety, that could only be conceived of or appreciated by those who had the happiness of his most intimate acquaintance, who enjoyed his endearing friendship, and who witnessed his labors, and he thus soon possessed himself of a collection of the most beautiful sections made by himself, and expensive illustrative preparations of every structure of the teeth that probable ever enriched the library or museum of a professional man.

Advancing still onward, in his honorable and persevering course, in January, 1839, he communicated a paper to the Medico-Chirurgical Society, on "The Structure, Physiology, and Pathology of the Persistent, Capsular Investments and Pulps of the teeth." The object of that paper was, to direct the attention of the anatomists, first, to the capsular investments on the surface of the enamel; secondly, to the layer external to the crusta petrosa; and thirdly, to the structure and development of the ossified pulp, which he considered to be a fourth constituent substance. The paper was received by the society with all the pleasure and approbation that could be due to a contribution from a gentleman of Mr. Nasmyth's high attainments.

In 1839, too, he gave to the world his "Researches on the Development, Structure, and Diseases on the Teeth." That work, besides containing much new and valuable matter, shows that he was most familiar with all the ancient and modern writings that have appeared on the subject treated by him. It likewise contains the most beautiful diagrams of the most perfect and interesting sections of the teeth that can be represented. It never was intended, however, by the author, to be a complete and full work on these important subjects but, so far as it goes, it is an imperishable production, dedicated to the author's brother, Mr. Robert Nasmyth, of Edinburgh, and almost as if he himself had designed that it should be handed down to posterity as a specimen of what might be said, strikingly, to show forth the humility and all but child-like simplicity of a great man's mind (and to which brother he only paid a well-merited compliment, for Mr. Nasmyth of Edinburgh, as has already been stated, is a man of the greatest experience, eminence and worth,) his own few and unsophisticated words of dedication are these:

"To Robert Nasmyth, Esq., *Edinburgh.*

"My Dear Robert:—As there is no one more capable than yourself of fully appreciating the beauty and importance of the subjects discussed in the following pages, and none so likely to regard their defects with an indulgent eye, it is to you that this work is dedicated, by

"Your Affectionate Brother,
Alexander Nasmyth.

"*London, George Street, Hanover Square,* May, 1839."

At the ninth annual meeting of "The British Association for the Encouragement of Science," held at Birmingham, in the month of August, 1839, Mr. Alexander Nasmyth read three memoirs: the title of the first was, "Investigations into the Structure of Fossil Teeth, &c.;" that of the second, "Investigation into the Development and Organization of the Dental Tissues, &c.,;" and that of the third, "Investigation into the Structure of the Epithelium."

These memoirs, though most instructive and interesting, and evidently the result of great research and observation, were not published by the Council of the Association in the regular way in which Mr. Nasmyth considered the transaction of that association ought to have been, and after evidently feeling that he had great cause to complain he says, "As many inquiries have been made for an authentic account of them, I think it best to publish the original materials of my communications as they were delivered at Birmingham, and I accordingly now proceed to lay before the public my papers, as they were there read." Accordingly, he himself published the memoirs, in one volume, under the title of "'Three Memoirs on the Development and Structure of the Teeth and Epithelium,' as read at the meeting at Birmingham." He has likewise accompanied those memoirs with many most beautiful diagrams, illustrative of his views.

As has been already stated, at this period, and long before, he was, and had been fully engaged in a most extensive practice, of the highest respectability, which required his unremitting personal attention, and the only wonder is, how a man occupied so fully in the practical part of such a business as he was, could possibly have found sufficient time to have proceeded, as he did, with his laborious investigations, and to have favored science with the fruits of them to the extent he did.

His character as a man of learning and profound knowledge in his profession was now fully established, and widely circulated, and in the year 18— he had the honor to be called upon to render his professional aid to his Royal Highness, Prince Albert; so highly did the Prince esteem his services and so favorable must the report of his Royal Highness have been of Mr. Nasmyth's abilities to the highest quarters, that very soon afterwards he received Her Majesty's commands to wait upon her, and to such an extent did she appreciate his professional attainments, that she distinguished him with an appointment to be her own Surgeon-Dentist, and to take the care and management of the royal children's teeth, and the duties of that high office he discharged with the utmost satisfaction to his royal employers to the last.

In 1843, a new charter was granted to the Royal College of Surgeons, London, by which the name of that college was changed to that of the "Royal College of Surgeons of England," by that charter, amongst other privileges, the degree of a *fellowship* was to be conferred upon certain of the members of that college, and as if sufficient honor had not been heaped upon the worthy man's head, whose course of life we are now reflecting upon. Mr. Nasmyth was amongst the first of the members upon whom the distinction of the fellowship was conferred by that distinguished and learned body, and which distinction he valued beyond all price.

Here then was a man selected and chosen by his sovereign and by her royal consort, to afford them and their children that professional aid, which they must have considered him to be the best qualified of all competitors to render, and then honored by the most competent of all persons to judge of his worth and abilities, by the highest compliment which the crown had enabled them to confer on a few of the most distinguished of their members.

Now at the time at which he was thus in the plentitude of his well earned honors and usefulness, he was laboriously employed in the preparation of a work on Odontology, of a most comprehensive character, requiring the deepest and most accurate research, the minutest investigations and the most extensive knowledge of the greatest variety of circumstances, but, alas! the pleasure he had promised to himself, that of leaving to others the copious result of many years exertion on his part, proved to be a task too gigantic for him, for under the labor and incessant anxiety the preparation of it occasioned to him, he suddenly sunk. The prosecution of his object deprived him of the repose absolutely necessary for the preservation of his health; and although often advised to desist from his efforts even for a time, yet he still clung to his studies as the object most dear to him, refusing to listen to the solicitations of his friends, and just as he was about to complete the MSS. of his great work, and looking forward to its speedy publication, but before he had time either to place it in his printer's hands or even to revise what he had written, it pleased that Providence to whose overruling control all human views and desires must bow, to decree that enough had been done by that excellent man—for retiring one night to his bed-chamber in his usual health, without making a complaint, and enjoying a night's good rest, on leaving his bed in the morning, and before he had yet either shaven or dressed himself, he was suddenly prostrated by an attack of apoplexy. He was attended without delay by Sir James Clarke, Her Majesty's Physician, and other medical friends of the first skill, and though he was not carried off on that occasion, yet hewas left so deprived of his former fine abilities, both mental and physical, as to require to be immediately removed from all excitement and intellectual exertion. He lived for about two years afterwards, and though he enjoyed tolerable bodily health, yet he was still in a very enfeebled state. In the course of that interval he visited his brother at Edinburgh, and after remaining with him a considerable time, he returned to London. Subsequently he went to Malvern, a place in Gloucestershire, greatly celebrated for its beautiful scenery and salubrious air, but he was only there for a very short period, when he sustained another visitation which abruptly closed his career, on the third day of August, 1849, thus dying in the 60th year of his age.

He has left a widow, but no children, and his widow has put together and published the MSS. of his intended Odontological work; but as he was not permitted to arrange the MSS. according to his own views, nor to correct or revise it in any way, nor at all to complete the preparation of it for the press, it would not be fair to his memory, that critics should regard the posthumous publication of the rough materials which he had prepared, even at the cost of his own life, as in all respects accurately containing his views precisely, embodying his facts and disclosing his novelties in the form, terms and manner, in which he intended they should be issued to the public, as a standard criterion by which his valuable attainments and abilities as a practitioner, philosopher and author, should be judged of.

It cannot be too deeply regretted also, that his most costly, and in many respects, rare and invaluable museum, library and collection of instruments and diagrams, should in the confusion into which through his sudden and unexpected illness, his widow permitted his matters and affairs to be plunged, have been scattered and lost, alike unprofitably to his estate and to the usefulness of his fellow laborers and his profession.

These, then, are the leading points in the history of the boy, the man, the professor, and the example in his character that has been left to us; in short, of the rise, progress and end of a man whose death the literary and scientific world must all mourn. From a comparatively humble condition in life, without in youth having been designed by his parents to fill any eminent position, and unfavored by any very peculiar advantages, by industry, probity, and the zealous improvement of his natural gifts, he rose over the heads of all his competitors, attracting the notice and the favors of royalty, to be the first practitioner in one of the most enlightened and necessary professions in that city, which may well be called, as it often is, the metropolis of the world. The whole of his history strikingly shows what may be achieved by those who may choose the good path, of applying themselves as he devoted himself; and who, after performing their different parts on this terrestrial stage may, like him, descend at the close of their labors, to their final resting places, beloved by those to whom they may be known, honored and esteemed by those to whom they could be known by reputation, and leaving an example behind them, worthy of all imitation.

